# Chronic kidney disease of uncertain aetiology: adding vital piece of information to the national project team report of Sri Lanka

**DOI:** 10.1186/s12882-015-0211-5

**Published:** 2015-12-23

**Authors:** T. B. Ananda Jayalal

**Affiliations:** Environmental Health & Occupational Health, Ministry of Health, Colombo, Sri Lanka

**Keywords:** Cadmium, Nephropathy, Chronic Kidney Disease of uncertain aetiology (CKDu), Provisional tolerable intake (PTWI)

## Abstract

In a recent study published by the National Project Team on chronic kidney diseases of unknown origin in Sri Lanka, identified cadmium as a major risk factor but strong conclusions were not made as the identified environmental toxins were within the permissible levels.

Sri Lankan food consumption pattern is different so that approach of total exposure of cadmium by food and water been calculated. Such calculation point out that total exposure of cadmium exceed the provisional tolerable weekly intake determined by international agencies.

## Background

It is been estimated more than 40,000 cases of chronic kidney disease patients in areas where chronic kidney disease of uncertain aetiology (CKDu) prevails in Sri Lanka. Large proportions of these cases are due to uncertain aetiology. National project team on chronic kidney diseases has published that chronic exposure to low levels of cadmium may be a causative factor in CKDu in Sri Lanka [1]. However, Jayatilake et al. finding on rice and pulses and water cadmium values seems to be not supporting strongly as a causative factor as none exceeds the permissible upper limits. Jayatilake et al. have shown maximum cadmium levels in rice is 100 μg/kg and maximum cadmium levels in water as 1.5 μg/L. The Codex upper limit of cadmium for polished rice is 400 μg/kg [2] whereas World Health Organization upper limit for cadmium for drinking water is 3 μg/L [3]. However, in certain vegetables and freshwater fish Jayatilake et al. has found levels above the international permissible levels [1]. Nevertheless, Jayatilake et al. have suspected people living in endemic area could be exposed to cadmium exceeding tolerable limits.

Presence of cadmium in environmental samples similar to Jayatilake et al. are found by number of independent researches. Jayalal [4] has quoted cadmium testing done on environmental samples by Rice Research institute of Sri Lanka, National Water Supply and Drainage Board of Sri Lanka. Further, Bandara et al. [5, 6] and Andrew A. Meharg et.al. [7] also have shown presence of considerably higher levels of cadmium in their samples.

From above findings it is apparent that in some areas soil, on which food crops are grown, is rich in cadmium, may be due to natural presence augmented by introduction of fertilizers. It had been shown fertilizer “triple superphosphate” contain considerable levels of cadmium as a contaminant. In addition, change of physical properties of soil such as temperature, pH, and organic content etc. may be playing a role in the increase absorption of cadmium in to the food crops grown in those areas.

It is the total exposure to any toxin that matters when health impacts are considered. In heavy metal exposure and other environmental contaminants Food and Agriculture Organization (FAO) recommends the total diet study approach. Total exposure from total food intake, water and smoking gives the required information for scientific evaluation of the question. Valuable findings of the Jayatilake et al. [1] can be further augmented by calculating the total exposure to cadmium by the study population.

In addition hot spot sampling in areas with greater reporting of CKDu prevalence could provide strong link to risk factors [8]. Average values may not lead to concrete evidence as done by Jayatilake et al. [1].

## Methods

Sri Lankan diet is mainly consisting of rice, vegetables and small amount of proteins from animal or aquatic origin. Food made out of wheat also take minor proportion of the staple diet. From the available data, for an average 60 kg Sri Lankan living in endemic area it is assumed following food consumption values per week. Three kg of rice, 1.4 kg of vegetables and drinks 14 L of water per week. Other food categories not considered here as cadmium contribution from them are proportionately negligible or data on cadmium are not available. The figures available in the cluster diets published by WHO [9] and data available from the ministry of agriculture [10] were used with modification in this model food consumption values.

## Results and discussion

If the mean values of cadmium generated for food items by Jayatilake et al. are used for calculation of total exposure, 3 kg of rice with 25 μg/kg Cadmium, 1.4 kg of vegetables with 50 μg/kg of cadmium, and 14 L of water with 0.5 μg/L of cadmium following total weekly exposure of 152 μg of cadmium per week is obtained. If the upper values i.e.100 μg, 70 μg, 1.5 μg for rice, vegetables, and water respectively taken in to consideration exposure of 419 μg of cadmium per week obtained. Therefore it can be assumed that 152 to 419 μg of cadmium exposure per week occurs in endemic area only through food and water. Smokers are exposed to additional dose. The provisional tolerable weekly intake for cadmium for US and Europe declared by the relevant authorities are 2.52 μg per Kg body weight [11, 12] and corresponding value declared by WHO is 5.8 μg per Kg [13]. This correspond with 151.2 μg and 348 μg per week respectively for a 60 kg man.

The calculated cadmium exposure values exceed the provisional tolerable weekly intake (PTWI) for cadmium for European standard and if the worst case scenario considered exceeds the WHO recommendation (which is higher) as well. As the smokers get added dose of cadmium the actual exposure should be higher than above values. The contribution from smoking cannot be assessed in Sri Lanka as the adequate data not available. However, it is obvious from this calculations that certain number of people living in cadmium hot spots are exposed to doses exceeding the recommended tolerable intake of cadmium.

## Discussion

A wealth of data had been generated by Jayatilake et al. [1] to design the public health interventions to reduce the CKDu prevalence. However, Jayatilake et al. have not come to firm conclusion as the food consumption pattern and total exposure to the possible nephrotoxins were not researched in to, perhaps it is not the objective of the research. The future research should fill this gap of knowledge. However, present knowledge can be utilized in effective public health interventions and operational research on such interventions may lead to firm establishment of causation of CKDu.

## Conclusions and recommendations

Above calculations show that Sri Lankan population living in certain geological regions are exposed to cadmium from food and water in excess of provisional tolerable weekly intake recommended by international agencies.

As the cost of managing large number of people with terminal renal failure and the societal effects of premature death and disability is detrimental, following interventions are proposed which seems to be cost effective. However, it is suggested to consider the cost benefits in depth which is beyond the scope of this paper.

Future research need to be focused to confirm or exclude the causation of CKDu by cadmium. Following options are suggested.

1. Study the cadmium concentration in live or postmortem kidney samples in CKDu and non CKDu cases.
2. Cohort study the urinary excretion of cadmium and suitable biomarker of kidney damage after appropriate intervention to lower the cadmium intake. e.g. after provision of cadmium free water.

In the present context what is required is implementation of public health interventions to reduce the cadmium exposure of the population of the endemic area. From the current data available, following measures can be recommended.

1. Drinking water of identified areas to be tested for cadmium and suitability for drinking should be recommended if cadmium cannot be detected by a test method with limit of detection (LOD) > 10^−9^/L.
2. A national level programme to reduce cadmium from the food chain need to be initiated with the involvement of the ministry of health and ministry of agriculture. Following interventions can be proposed from the current knowledge.
  - All the major food varieties to be tested for cadmium and how cadmium concentrations varies with the food type and geographical variation need to be identified. Rice varieties with low cadmium concentration or best fields for paddy cultivation need to be identified and agronomic practices that reduce cadmium concentration in rice and other major food varieties to be popularized among farmers.
  - Anthropogenic activities which lead to increase cadmium levels in soil and food should be minimized to the extent possible.
  - Proper disposal mechanism for e waste which lead to decrease of cadmium concentration in the environment need to be implemented.
  - More polished rice to be recommended for vulnerable groups of population while addressing the micronutrient requirement as polishing had been shown low cadmium in rice.
3. To revise the Sri Lanka standard of potable water (SLS 614) and bottled or packaged drinking water and bottled packaged natural mineral water in the present context. The permissible levels for cadmium can be lowered to undetectable by a sensitive test method. (Graphite Atomic absorption or more sensitive method)
4. Coordinating mechanism on future research need to be implemented.

## Abbreviations

CKDu: chronic kidney disease of uncertain aetiology
FAO: Food and Agriculture Organization of United Nations
PTWI: provisional tolerable weekly intake
WHO: World Health Organization

## References

[1] Jayatilake N, Mendis S, Maheepala P, Mehta FR, On behalf of the CKDu National Research Project Team (2013). Chronic kidney disease of uncertain aetiology: prevalence and causative factors in a developing country. BMC Nephrol.

[2] FAO/WHO. Codex General Standard for Contaminants and Toxins in Food and Feed. Food and Agriculture Organizaton of the United nation, Joint FAO/WHO Food Standards Programme, Codex Alimentarius Commission. Rome: CODEX STAN; 2013. p. 193–1995.

[3] World Health Organization: Guidelines for drinking-water quality, fourth edition 2011. http://www.who.int/water_sanitation_health/publications/2011/9789241548151_ch08.pdf?ua=1(2011). Accessed 31 July 2015.

[4] Jayalal TBA (2015). Chronic exposure to nephrotoxic doses of Cadmium through Sri Lankan diet. Sri Lanka J Med Admin.

[5] Bandara JMRS, Senevirathna DMAN, Dasanayake DMRSB, Herath V, Bandara JMPR, Abeysekara T (2008). Chronic Renal failure among farm families in cascade irrigation systems in Sri Lanka associated with elevated dietary cadmium levels in rice and freshwater fish (Tilapia). Environ Geochem Health.

[6] Bandara JMRS, Wijewardana HVP, Liyanage J, Upul MA, Bandara JMUA (2010). Chronic renal failure in Sri Lanka caused by elevated dietary cadmium: Trojan horse of the green revolution. Toxicol Lett.

[7] Meharg AA, Norton G, Deacon C, Williams P, Adomako EE, Price A (2013). Variation in rice cadmium related to human exposure. Environ Sci Technol.

[8] Jennifer Redmon H, Elledge MF, Womack DS, Wickremashinghe R, Wanigasuriya KP, Peiris John RJ (2014). Additional perspectives on chronic kidney disease of unknown aetiology (CKDu) in Sri Lanka – lessons learned from the WHO CKDu population prevalence study. BMC Nephrol.

[9] World health Organization, Global Environment Monitoring System (GEMS/Food) http://www.who.int/nutrition/landscape_analysis/nlis_gem_food/en/(2012). Accessed 20 Dec 2015.

[10] Agriculture and Environment Statistics Division, Department of Census and Statistics, Sri Lanka. (2012) http://www.statistics.gov.lk/agriculture/Paddy%20Statistics/PaddyStatsPages/SelfsufficiencyRateofRice.html. Accessed 31 July 2015.

[11] Tox Guide for Cadmium. U.S. Department of Health and Human Services, Public Health Service, Agency for Toxic Substances and Disease Registry. 2012. http://www.atsdr.cdc.gov/toxguides/toxguide-5.pdf (2012). Accessed 31 July 2015.

[12] EFSA CONTAM Panel (EFSA Panel on Contaminants in the Food Chain), 2009. Cadmium in food - Scientific opinion of the Panel on Contaminants in the Food Chain. doi:10.2903/j.efsa.2009.980 (http://www.efsa.europa.eu/en/efsajournal/pub/980) 2011, Accessed 31 Jan 2015.

[13] FAO/WHO (2011). Safety evaluation of certain food additives and contaminants, joint FAO/WHO expert committee on food additives (JEFCA).

